# Water-Dispersible Carboxymethyl Dextran-Coated Melamine Nanoparticles for Biosensing Applications

**DOI:** 10.1021/acsomega.2c05653

**Published:** 2022-11-03

**Authors:** Yoshikazu Kurihara, Hiroyuki Yokota, Masaru Takahashi

**Affiliations:** Konica Minolta, Inc., 1 Sakura-machi, Hino-shi, Tokyo 191-8511, Japan

## Abstract

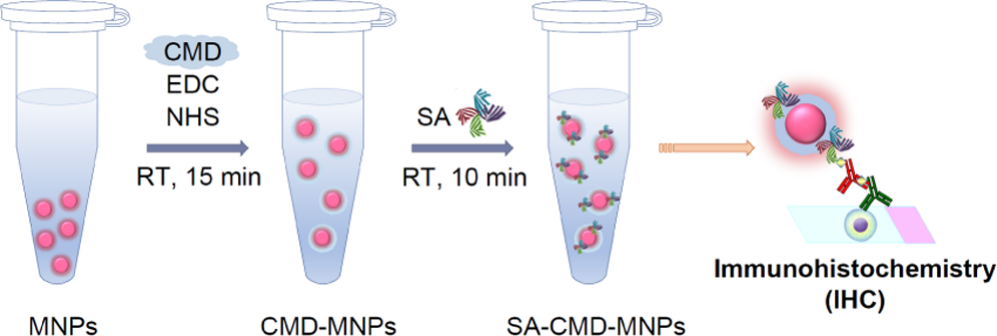

In this study, we developed a simple method for preparing highly dispersed, stable, and streptavidin (SA)-functionalized carboxymethyl dextran (CMD)-coated melamine nanoparticles (MNPs) in an aqueous buffer at neutral pH. Dynamic light scattering (DLS) revealed the agglomeration of MNPs in an aqueous buffer at neutral pH. When CMD, *N*-hydroxysuccinimide (NHS), and 1-ethyl-3-(3-dimethylaminopropyl) carbodiimide (EDC) were simultaneously mixed with the MNPs, CMD was bound to the MNPs, promoting their dispersibility. Preparation of SA-CMD-MNPs was accomplished simply by adding SA solution to the CMD-MNPs. The amount of SA bound to the CMD-MNPs was quantified by the bicinchoninic assay, and the amount of SA molecules bound to each CMD-MNP was 417 ± 4. SA-CMD-MNPs exhibited high dispersity (polydispersity index = 0.058) in a neutral phosphate buffer and maintained it for 182 days with dispersion using a probe sonicator (5 s) before DLS characterization. The performance of the SA-CMD-MNPs in biosensing was evaluated by immunohistochemistry, which revealed that the nanoparticles could specifically stain MCF-7 cells derived from breast cancer cells with low HER2 expression. This study provides an effective method for synthesizing highly dispersible nanoparticles for biosensing.

## Introduction

Nanomaterials permit unique approaches owing to their high surface-area-to-volume ratio compared with conventional fine particles and bulk materials.^1^ Thus, recently, nanomaterials have been widely employed in various fields, such as medical,^1−3^ electronics,^4^ and energy.^5,6^ Nanotechnology has progressed remarkably, especially in the medical field, and it is being employed in various applications, such as cancer detection, viral and bacterial detection by biosensing, visualization of disease sites by bioimaging, contrast agents for magnetic resonance imaging, and drug delivery for cancer treatment.

Various nanoparticles (NPs) have been reported for biomedical applications. In addition to metallic materials, such as iron oxide,^7^ gold nanoparticles,^8^ and ferrites,^9^ and quantum dots (QDs), such as cadmium selenide^10^ and indium phosphide,^11^ inorganic nanomaterials, such as carbon QDs,^12^ carbon nanotubes,^13^ and mesoporous silica,^14^ and organic nanomaterials composed of amphiphilic lipids, such as liposomes^15^ and dendrimers,^16^ are used to bind and detect target biomolecules.

However, several challenges hinder the development of these materials. One is the effective conjugation of biomolecules, such as peptides, oligonucleotides, antibodies, and proteins, to the surface of nanomaterials.^17−21^ When biomolecules bind to nanomaterials, the nanomaterials acquire the ability to bind to or sense specific locations, such as organs, tissues, cells, and organelles. Known as an avidin–biotin system, which has a very high affinity (*K*_d_ ∼10^–15^) to avidin and biotin,^22^ nanomaterials exhibit sensing properties and are used in bioassays for immobilizing either avidin or biotin.

Farka et al. synthesized photon-upconversion nanoparticles composed of NaY_0.895_Yb_0.10_Er_0.005_F_4_ modified with streptavidin (SA) based on click chemistry and employed them in the immunoassay of the honeybee pathogen *Melissococcus plutonius*(23) and HER2 immunocytochemistry.^24^ SA-coated polyurethane-urea nanoparticles prepared with O/W nanoemulsions via interfacial condensation have been employed for fluorescence imaging and targeting of tumor environments.^25^ Solid–lipid nanoparticles prepared by physical adsorption of SA and combination with a biotin-conjugated compact antibody have been employed for targeting human breast cancer cells,^26^ and gold nanoparticles with SA are used for immunoassay using lateral-flow test strips.^27^ Biotin-coated liposomes, which contain biotin-modified lipids, have also been reported. Liposomes are used for labeling and targeting human epidermoid carcinoma lines overexpressing EGFR^28^ or targeting biotinylated anti-CD16/32 on J774.1 cells.^29^

Although avidin and biotin have been loaded onto nanoparticles via click chemistry, physisorption, or incorporation into constituent materials, such as liposomes, bioconjugation is commonly performed via covalent bonding using a primary or secondary amino, carboxyl, hydroxyl, alkyl halogen, or azide group with very high binding strength.^7,11^ Commercially available reagents, such as 1-ethyl-3-(3-dimethylaminopropyl) carbodiimide (EDC), diisopropyl carbodiimide (DIC), *N*-hydroxysuccinimide (NHS) esters, amide coupling using condensing agents, such as [*N*,*N*,*N*′,*N*′-tetramethyl-O-(1H-benzotriazol-1-yl)uronium hexafluorophosphate] (HBTU) and *N*-succinimidyl 3-[2-pyridyldithio]-propionate (SPDP), and sulfosuccinimidyl-(4-*N*-maleimidomethyl)cyclohexane-1-carboxylate (sulfo-SMCC), are also effective in converting amino group ends to thiol or maleimide groups using bifunctional cross-linkers.^7^

The dispersity of nanoparticles in aqueous solutions is another challenge. Nanoparticles are prone to agglomeration due to their high surface energy. Therefore, to fully demonstrate the functions of nanoparticles, it is important to develop technologies to suppress the agglomeration/aggregation and improve the dispersibility of nanoparticles. Jiang et al. reported that the ionic strength, pH, and surface modification of particles significantly affect the ζ-potential and hydrodynamic diameter of TiO_2_ and QDs in water.^30^ Lartigue et al. synthesized iron oxide nanoparticles that disperse well in water and can serve as contrast agents or mediators for magnetic thermotherapy by covalently immobilizing monosaccharides, such as rhamnose.^31^ In addition, the use of amphiphilic polymers in preparing water-soluble nanoparticles^32,33^ and the use of carboxyl group-terminated compounds affect water solubility.^34,35^ Several studies have been conducted to improve the dispersibility of Fe nanoparticles by modifying carboxymethyl dextran (CMD) among carboxyl groups.^36−39^ Although nanoparticles with improved functionalities or dispersibility have been achieved, obtaining nanoparticles with both functionalities and dispersibility is still a challenge to overcome. In our previous study, Gonda et al. developed fluorescent melamine nanoparticles (MNPs) and investigated their application in the immunohistochemistry (IHC) of pathological tissues.^40^ They used a polyethylene glycol cross-linker for preparing SA-modified MNPs with both functionalities and dispersibility. But it required a very complicated process, and the mechanism was unclear.

**Figure 1:**
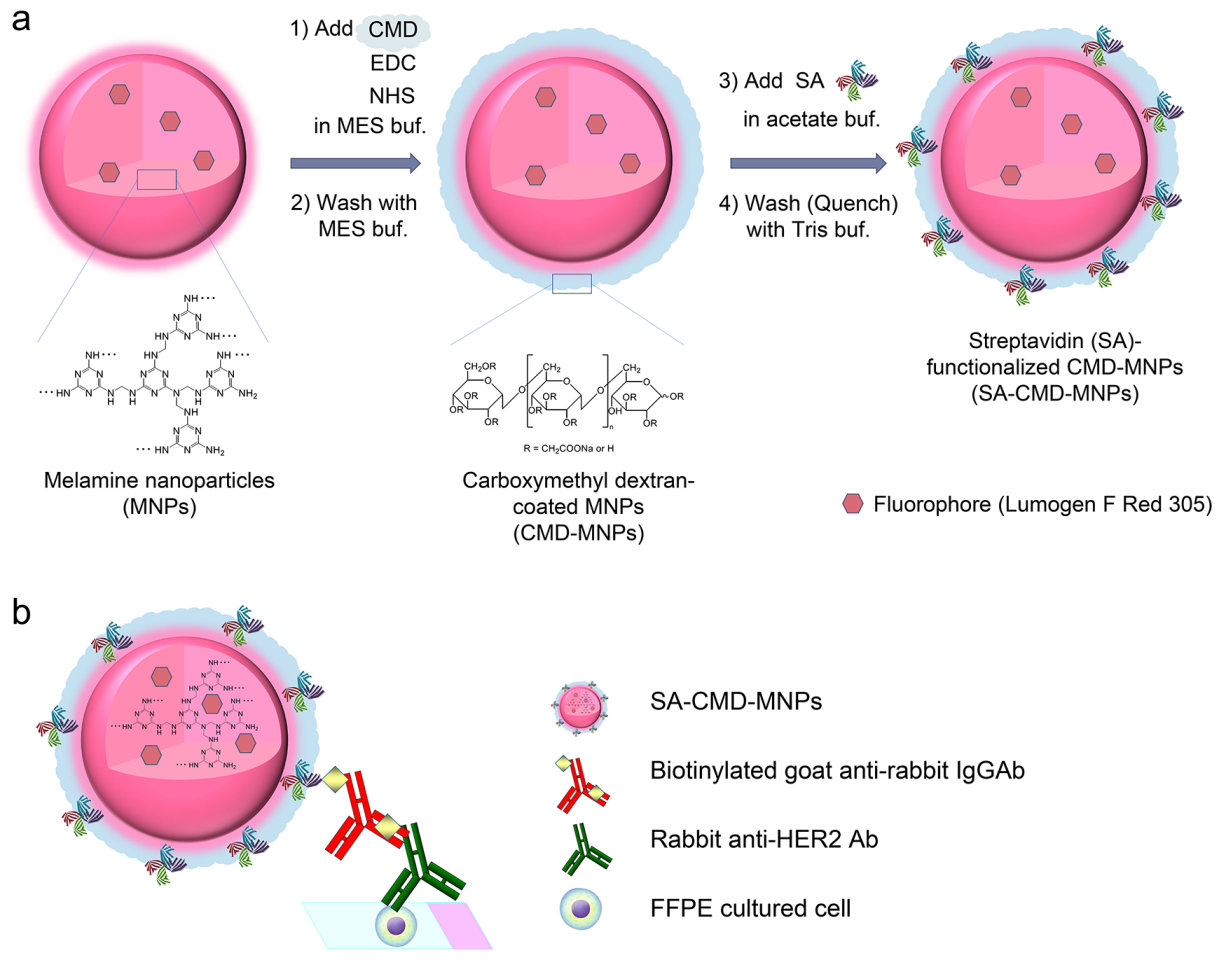
Schematic of (a) the synthesis of streptavidin-containing carboxymethyl dextran-coated melamine nanoparticles (SA-CMD-MNPs) and (b) the IHC assay using SA-CMD-MNPs as biosensors.

Methods that produce nanoparticles via clear, simple, and dispersible-ensuring mechanisms are strongly desired. We investigated the use of CMD for the surface modification of MNPs to improve their dispersibility and add functionality as biosensors. The dispersibility of MNPs was improved in a single step by simultaneously mixing CMD with NHS and EDC, which are commonly used as amide coupling agents (Figure 1a). After washing with MES buffer, SA was added to the CMD-coated nanoparticles (CMD-MNPs) to modify SA by binding to the remaining NHS ester in the CMD. Three well-known methods (bicinchoninic (BCA), Lowry, and Bradford) were employed to evaluate the amount of SA binding to MNPs. The significance of the reagents (CMD, EDC, and NHS) and buffer for the CMD/SA coupling was also investigated. The function of SA-CMD-MNPs as biosensors was evaluated by IHC using four types of formalin-fixed paraffin-embedded (FFPE) cultured cells (ZR-75-1, T-47D, MCF-7, and HT-1080) with different levels of HER2 expression (Figure 1b).

## Experimental Section

### Chemicals and Reagents

Emulgen (Kao Corporation, Tokyo, Japan), 1,3-bis(hydroxymethyl)urea (Tokyo Chemical Industry Co., Ltd., Tokyo, Japan), methylol melamine (Nippon Carbide Industries Co., Inc., Tokyo, Japan), dodecylbenzenesulfonic acid (Kanto Chemical Co., Inc., Tokyo, Japan), Lumogen F Red 305 (BASF AG, Ludwigshafen, Germany), dodecylbenzenesulfonic acid sodium salt (Kanto Chemical), CMD (CMD-L, molecular weight: 10 kDa, Meito Sangyo Co., Ltd., Nagoya, Japan), NHS (FUJIFILM Wako Pure Chemical Corporation, Osaka, Japan), EDC (Tokyo Chemical Industry), SA (ProSpec-Tany TechnoGene Ltd., Ness Ziona, Israel), rabbit anti-HER2 IgG (clone: EP1045Y, abcam, Cambridge, U.K.), biotinylated anti-rabbit antibody (DISCOVERY Universal Secondary Antibody, F. Hoffmann-La Roche Ltd., Basel, Switzerland), horseradish peroxidase-labeled anti-rabbit antibody (Histofine Simple Stain MAX PO (MULTI), Nichirei Biosciences Inc., Tokyo, Japan), 3,3′-diaminobenzidine (DAB, FUJIFILM Wako Pure Chemical), and all other reagents were analytical grade and used without further purification. Ultrapure water (UPW) used during the experiment was produced using a Millipore Milli-Q water purification system (Burlington, MA).

### Preparation of MNPs

First, 29 mg of Emulgen was dissolved in 18.2 mL of UPW. Then, the solution was stirred and heated at 70 °C. Next, 570 μL of 5 wt % 1,3-bis(hydroxymethyl)urea, 200 μL of 50 wt % methylol melamine, and 1 mL of 5 wt % dodecylbenzenesulfonic acid aqueous solution were added and maintained at 70 °C for 1.5 h. After washing with UPW, seed particles were obtained. Next, 850 μL of the seed particle solution, 1 mL of 1 wt % sulfonated Lumogen F Red 305, 800 μL of 5 wt % Emulgen, and 1 mL of 1.7 wt % dodecylbenzenesulfonic acid sodium salt aqueous solution were added to UPW (15 mL). Then, the solution was stirred and heated at 70 °C. Next, 420 μL of 50 wt % methylol melamine and 1 mL of 50 wt % dodecylbenzenesulfonic acid/*p*-toluenesulfonic acid aqueous solution were added and heated at 70 °C for 50 min. Then, the temperature was increased to 90 °C and maintained for 20 min. After centrifuging the solution (4 °C, 20 000*g*, 20 min), the supernatant was removed, and 10 mL of UPW was added to the pellet. The MNPs were dispersed using a probe sonicator (UH-50, SMT Co., Ltd., Tokyo, Japan).

### Preparation of SA-CMD-MNPs

First, 500 μL of CMD (6 mg/mL) in an MES (pH 5.0) buffer, 117.5 μL of MNPs (final concentration: 4.73 nM) aqueous solution, 250 μL of NHS (100 mM) in MES, and 250 μL of EDC (400 mM) in MES were added to a 2 mL microtube. The mixed solution was stirred using a rotator for 15 min at room temperature. After centrifuging the solution (4 °C, 20 000*g*, 20 min), the supernatant was removed, and 1 mL of MES was added. The pellet was dispersed using a probe sonicator for 5 s. After repeating centrifugation, supernatant, removal, MES addition, and dispersion, a portion of the solution (200 μL) was sampled (sample A). The solution was centrifuged, and the supernatant was removed from the solution. After adding 680 μL of acetate buffer (pH 6.0) and 120 μL of SA solution (1 mg/mL) to the pellet, the solution was dispersed using a probe sonicator for 5 s and stirred using a rotator for 10 min at room temperature. Then, it was centrifuged (4 °C, 20 000*g*, 20 min), the supernatant was removed, and 800 μL of Tris buffer (pH 8.5) was added. The pellet was dispersed using a probe sonicator for 5 s. After repeating centrifugation, supernatant, removal, Tris addition, and dispersion, a portion of the solution (200 μL) was sampled (sample B). The solution was centrifuged, and the supernatant was removed from the solution. After adding 600 μL of a blocking buffer (3.0 wt % bovine serum albumin, 1.2 wt % casein (Sigma) in Tris buffer (pH 7.6)) to the pellet, the solution was dispersed using a probe sonicator for 5 s. The MNP solution in the blocking buffer was designated as sample C and used for IHC.

### Characterization of MNPs, CMD-MNPs, and SA-CMD-MNPs

The morphologies of the MNPs, CMD-MNPs, and SA-CMD-MNPs, the particle sizes of the MNPs, and their distribution were evaluated using an SEM (S-4800, Hitachi High-Tech Corp., Tokyo, Japan). The excitation and fluorescence emission spectra of MNPs, CMD-MNPs, and SA-CMD-MNPs were measured using a fluorescence spectrophotometer (F-7100, Hitachi High-Tech). The Fourier transform infrared (FT-IR) spectra of MNPs, CMD-MNPs, and SA-CMD-MNPs were obtained using an FT/IR-4600 spectrometer (JASCO Corp., Tokyo, Japan) and an IRT-5200 FT-IR microscope (JASCO). Each nanoparticle pellet was pressed with a diamond compression cell on an aluminum-coated slide, and the IR measurements were conducted in a reflection mode with a 16× Cassegrain objective lens.

The *Z*-average, PdI, and ζ-potential of MNPs, CMD-MNPs, and SA-CMD-MNPs were measured by DLS (Zetasizer Nano ZS, Malvern Panalytical Ltd., Malvern, U.K.) with disposable folded capillary cells. MNPs, CMD-MNPs, and SA-CMD-MNPs were diluted 50-fold (CMD-MNPs or SA-CMD-MNPs) or 500-fold (MNPs) in a 10 mM phosphate buffer (pH 7.2) and used for the measurement under a neutral condition. When the pH dependence of MNPs and SA-CMD-MNPs was evaluated, 10 mM KCl–HCl buffer (pH 2), 10 mM acetate buffer (pH 4 and 5), 10 mM phosphate buffer (pH 6 and 12), and 10 mM borate buffer (pH 9.8) were also used for the measurement. When the stability of SA-CMD-MNPs was evaluated, both the solution dispersed by 5-s probe sonication before characterization and the solution allowed to stand were used.

BCA (Thermo Fisher Scientific, Waltham, MA), Lowry (Thermo Fisher Scientific), and Bradford (Dojindo Laboratories, Kumamoto, Japan) were used to measure protein concentration according to the manufacturer’s instructions. To calculate the amount of protein bound to SA-CMD-MNPs, a calibration curve, which shows the relationship between SA concentration and absorbance, was obtained using the BCA assay. The absorbance of samples A (CMD-MNPs) and B (SA-CMD-MNPs) was measured, and the difference was fitted to the calibration curve.

### Cell Culture and Preparation of FFPE Cell Blocks

The ZR-75-1, T-47D, MCF-7, and HT-1080 cells were obtained from American Type Culture Collection (ATCC, Manassas, VA). Each cell line was cultured in a T225 culture flask and according to ATCC’s recommendations. When the cells reached 80% confluency, they were treated with trypsin/EDTA and trypsin neutralization solution. After the cells were washed twice with PBS (pH 7.4), they were fixed with 10% neutralized formalin at 4 °C overnight. Then, the cells were washed again with PBS. Thereafter, the cells were suspended in a 1 wt % sodium alginate solution and gelled with 1 M calcium chloride solution. The paraffin cell blocks were arrayed into a single paraffin recipient block using a Tissue Micro Array Set (Labro Co., Ltd., Seoul, Korea). The single-cell block array was sectioned at 4 μm using a sliding microtome (REM-710, Yamato Kohki Industrial Co., Ltd., Saitama, Japan).

### IHC

SA-CMD-MNPs were characterized using a BOND RX autostainer (Leica Biosystems). Following dewax and rehydration, the FFPE slides underwent antigen retrieval at pH 9 for 40 min, and the blocking buffer used for the SA–CMD-MNPs was used for protein blocking. The slides were incubated with rabbit anti-HER2 IgG diluted at 1:400 in the blocking buffer for 1 h. In a negative control experiment, the slides were incubated in the same dilution buffer without the anti-HER2 antibody. Then, biotinylated anti-rabbit antibody diluted at 1:10 in the blocking buffer was applied for 30 min. Thereafter, the slides were incubated with SA-CMD-MNPs (0.16 nM) for 2 h, 4% paraformaldehyde for 10 min, and Mayer’s hematoxylin for 5 min. For HER2 immunostaining with DAB, one DAB tablet was dissolved in 50 mM Tris-HCl buffer (pH 7.6, 50 mL) and 30% hydrogen peroxide water (10 μL) to prepare a DAB solution. After dewax, rehydration, antigen retrieval, and blocking as previously described, the slides were incubated with the horseradish peroxidase-labeled anti-rabbit antibody instead of the biotinylated anti-rabbit antibody, followed by DAB (5 min) and Mayer’s hematoxylin (5 min). Finally, the slides were washed with distilled water, and a glass coverslip was mounted using malinol and an automated glass cover slipper (Tissue-Tek Glas g2, Sakura Finetek). The SA-CMD-MNPs fluorescence was imaged using a fluorescence microscope (BX53, Olympus Corporation, Tokyo, Japan) with a UPLSAPO 40 × 2 (Olympus) objective lens and a CCD camera (DP80, Olympus).

## Results and Discussion

### Characterization of MNPs, CMD-MNPs, and SA-CMD-MNPs

**Figure 2:**
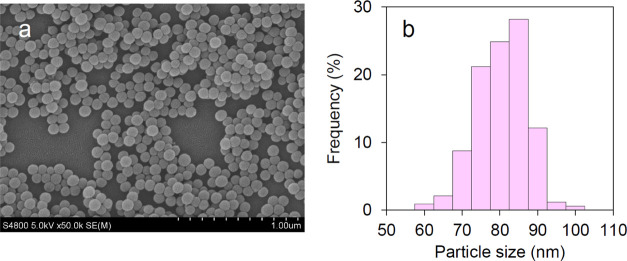
(a) Scanning electron microscopy (SEM) image and (b) particle size distribution of MNPs.

The MNPs were characterized by scanning electron microscopy (SEM). The MNP microstructures were almost perfectly spherical (Figure 2a), and the particles were uniform with an average particle size of 83 ± 7 nm (Figure 2b). The morphology of the MNPs was unchanged after modification (Figure S1). The properties of the encapsulated fluorophore were reflected in the excitation and fluorescence emission spectra of the MNPs. The fluorescence properties of the CMD-MNPs and SA-CMD-MNPs were almost identical to those of the MNPs (Figure S2). These results indicate that the modification conditions of CMD and SA on the MNPs are mild and affect neither the morphology of the nanoparticles nor the encapsulated fluorophores.

The structure of the obtained nanoparticles was evaluated by FT-IR (Figure S3). The FT-IR spectral band assignments based on literature data are listed in Table S1.^41,42^ All bands were derived from the melamine formaldehyde resin, indicating that the nanoparticle composition is almost exclusively melamine. Three bands from 1300 to 1600 cm^–1^ (methylene-derived C–H bending (1348, 1492 cm^–1^) and triazine-derived C=N stretching (1561 cm^–1^)) in the spectrum of the MNPs were shifted to shorter wavenumbers after modification with CMD (C–H bending (1345, 1488 cm^–1^) and C=N stretching (1556 cm^–1^)), indicating that hydrogen bonding caused by the carboxyl and hydroxyl groups of CMD weakened the interatomic bonding.^43^ Subsequently, immobilization of SA shifted the band toward longer wavenumbers (C**–**H bending (1352, 1504 cm^–1^) and C=N stretching (1580 cm^–1^)) from those of the original MNPs (Figure S3b). These shifts might arise from strong electrostatic interactions between the charged amino acid residues on the SA surface and the melamine resin.

**Figure 3:**
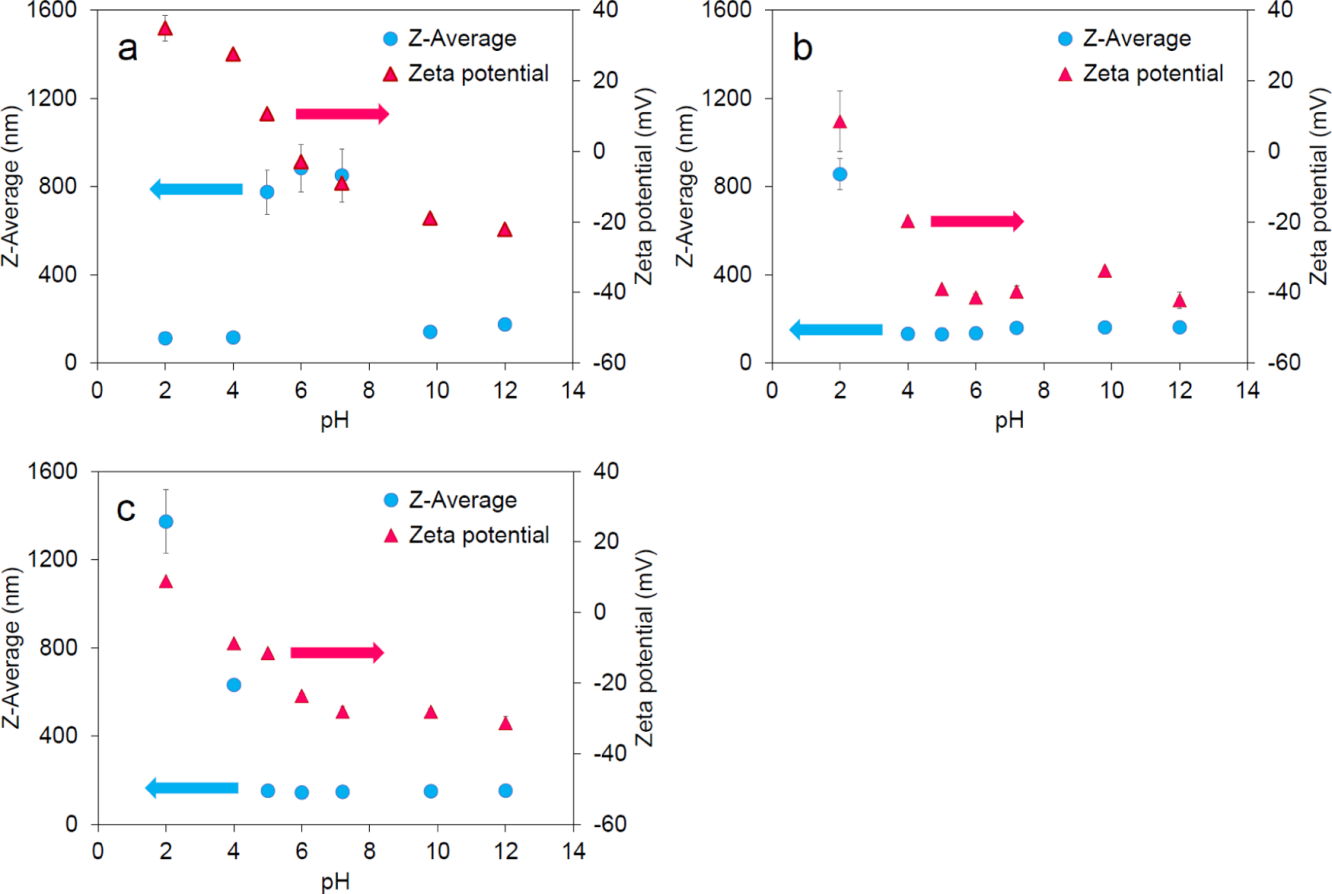
*Z*-average and ζ-potential of (a) MNPs, (b) CMD-MNPs, and (c) SA-CMD-MNPs.

Before surface modification, the MNPs were highly dispersive in acidic solutions at pH 2 or 4, with PdI of 0.029 and 0.021, respectively (data not shown). The hydrodynamic diameters (*Z*-average) were 112 and 116 nm (Figure 3a). PdI less than 0.1 indicates high dispersibility,^44^ and consistent with the SEM results, the nanoparticles showed very high dispersibility in aqueous solutions, depending on the conditions.

The reason for the larger particle size observed by dynamic light scattering (DLS) than that observed by SEM may be that DLS measures the hydrodynamic diameter, which includes the hydration shell.^45^ The isoelectric point (pI) was between pH 5 and 6, and the MNPs agglomerated and became moderately polydisperse in the weakly acidic to neutral range. On the other hand, CMD-MNPs and SA-CMD-MNPs exhibited dispersibility over a wide pH range, from weakly acidic to alkaline (Figure 3b,c). This indicates that MNPs do not exhibit sufficient dispersibility in biosensing assays that often use near-neutral buffers, which can be overcome by modifying them with CMDs.

**Figure 4:**
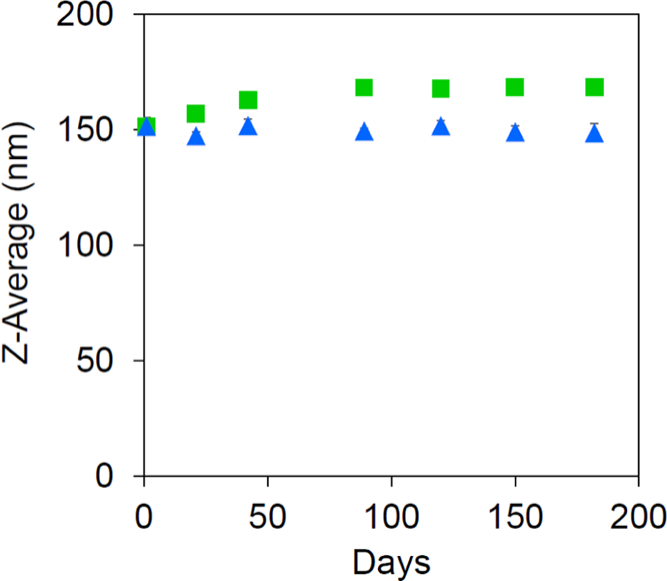
Stability of SA-CMD-MNPs with time. The green squares represent the *Z*-average of SA-CMD-MNPs incubated at 4 °C, and the blue triangles represent that of SA-CMD-MNPs dispersed by probe sonication before dynamic light scattering (DLS).

The SA-CMD-MNPs maintained their particle sizes (147–152 nm) for 182 days of dispersion with probe sonication before characterization (Figure 4). The particle size slightly increased when the particles were refrigerated, which is not a result of the breakage of strong bonds, such as covalent bonding, but weak interactions, such as electrostatic interactions, intermolecular forces, and hydrogen bonds, which caused the agglomerate of particles.

**Figure 5:**
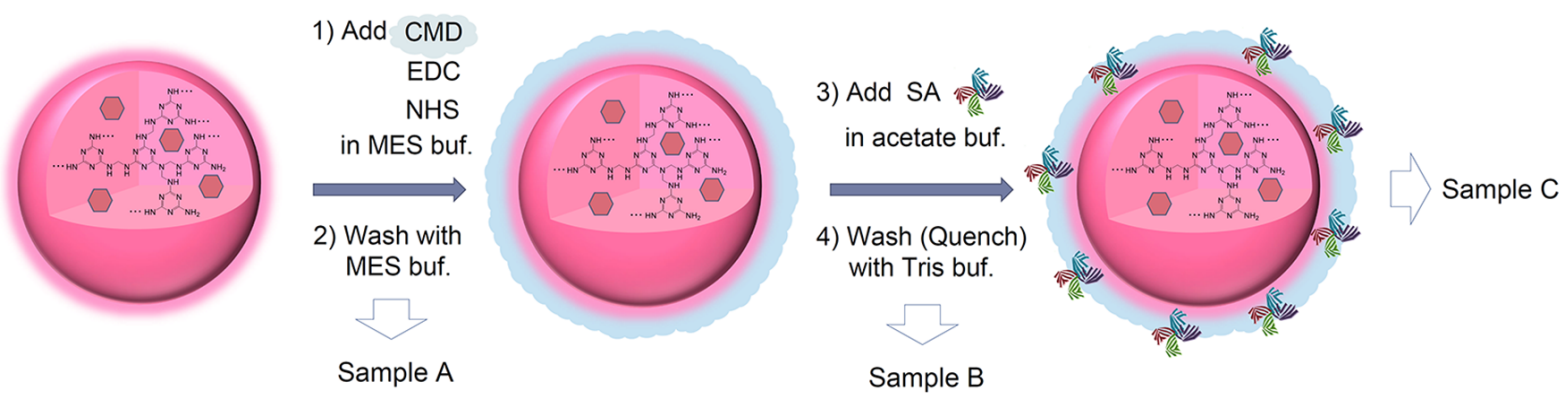
Schematic of the validation system. Samples A and B were obtained from MNPs in (2) and (4) wash buffer, respectively, and sample C was obtained from the blocking buffer. The samples were diluted at 1:100 in phosphate buffer (PB, pH 7.2) and evaluated by DLS.

The importance of CMD, NHS, and EDC reagents and buffer selection in the preparation of CMD-SA-MNPs was evaluated by sampling MNPs in the process of preparation (Figure 5). Table 1 shows the conditions under which the CMD-SA-MNPs were prepared under different conditions (Nos. 1–8), and Table 2 shows the results of DLS measurement of each MNP.

**Table 1:** Reaction Conditions Employed for SA-CMD-MNP Preparation, Showing the Effects of CMD, 1-Ethyl-3-(3-dimethylaminopropyl)carbodiimide (EDC), and *N-*Hydroxysuccinimide (NHS) Reagents and Buffer Selection

	no.							
process	1	2	3	4	5	6	7	8
(1) CMD	+	–	+	+	+	+	+	+
(1) NHS	+	+	–	+	+	+	+	+
(1) EDC	+	+	+	–	+	+	+	+
(1) and (2) reaction and wash buffer	MES	MES	MES	MES	PB	MES	MES	MES
(3) SA	+	+	+	+	+	–	+	+
(3) reaction buffer	acetate	acetate	acetate	acetate	acetate	acetate	PB	acetate
(4) wash buffer	Tris	Tris	Tris	Tris	Tris	Tris	Tris	PB

**Table 2:** ζ-Potential, *Z*-Average, and PdI of SA-CMD-MNPs Synthesized Under Eight Reaction Conditions

no.	sample	ζ-potential (mV)	*Z*-average (nm)	PdI
1	A	–38.9	167	0.045
	B	–29.3	164	0.037
	C	–26.7	151	0.058
2	A	–11.1	1870	0.32
	B	–8.9	1000	0.27
	C	–26.6	144	0.065
3	A	–34.2	207	0.079
	B	–35.0	160	0.023
	C	–29.0	148	0.036
4	A	–27.5	128	0.069
	B	–21.2	834	0.29
	C	–27.4	136	0.037
5	A	–41.9	429	0.44
	B	–34.4	147	0.039
	C	–29.4	141	0.068
6	A	–39.3	172	0.045
	B	–36.2	159	0.031
	C	–33.1	148	0.062
7	A	–39.5	164	0.053
	B	–30.2	157	0.034
	C	–27.8	152	0.061
8	A	–39.8	170	0.029
	B	–32.3	164	0.068
	C	–29.0	149	0.072

When CMD was bound to MNPs, the ζ-potential at pH 7.2 shifted to −38.9 mV, and PdI was 0.045 (Table 2). Notably, the particle size remained almost the same (or slightly smaller) as the step progressed. When CMD and SA were bound to MNPs, the mass per particle increased. These results are attributed to the increase in the dispersibility associated with the change in ζ-potential at neutral pH, as mentioned for the CMD-binding step before surface modification (Figure 3c). In addition, they are attributed to the consumption of the hydrophobic NHS ester remaining after CMD binding for the SA (+Tris)-binding step to the CMD support. The step of SA (+Tris) binding to the CMD support could be influenced by the dissolution of hydrophobic interactions between the MNPs due to the consumption of the hydrophobic NHS ester^46^ remaining after CMD binding and the dissolution of agglomeration due to the hydrophilicity of the protein.

When EDC and NHS were added without CMD (Table 1), the ζ-potential remained almost the same, and the agglomeration state was unchanged (Table 2). This indicates that CMD induced negative charges of the surface. The dispersion of particle size was not improved after the addition of EDC, NHS, SA, and Tris, except in the blocking buffer, indicating that the physical adsorption of BSA and casein in the blocking buffer improved the particle dispersibility. The presence of either NHS or EDC when CMD was added resulted in a significantly negative ζ-potential (Table 2). This is because the MNPs were positively charged at pH 5 (Figure 3a), whereas the pI of the carboxyl group of CMD was ∼4;^47^ therefore, CMD was negatively charged in the MES buffer at pH 5, and electrostatic adsorption occurs. In the system with CMD and NHS but not EDC (Table 2), CMD probably did not stay on the surface of the MNPs and was washed off by the Tris buffer during or after the addition of SA; thus, the MNPs were not dispersed but agglomerated after the SA reaction. In the system with CMD and EDC without NHS (Table 2), the particle size and dispersibility were similar to those of the system with CMD, EDC, and NHS after the addition of both CMD and SA, and the ζ-potential was almost unchanged after the addition of SA (from −34.2 to −35.0 mV). This is because, although amide coupling could occur with EDC alone,^48^ the coupling efficiency was insufficient, and although CMD could be immobilized, SA could not be immobilized. The activation efficiency of NHS and EDC decreases when the reaction buffer for CMD, NHS, and EDC changes from MES to phosphate buffer.^49,50^ Possibly, the dispersibility of MNPs after the CMD reaction was also insufficient due to the inefficient reaction efficiency of NHS and EDC in phosphate buffer (Table 2). After the SA reaction, both the agglomeration and dispersibility were improved, and the obtained particles were smaller than those obtained under standard conditions. The ζ-potential did not change much when SA was not added (Table 2), which is because Tris has only hydroxyl groups at the end other than the amino group, and Tris reactions with NHS esters do not affect the ζ-potential change, whether it is bound or not. Particle size, dispersibility, and protein binding were not significantly affected when phosphate buffer was used during the SA reaction and Tris quenching (Table 2).

**Figure 6:**
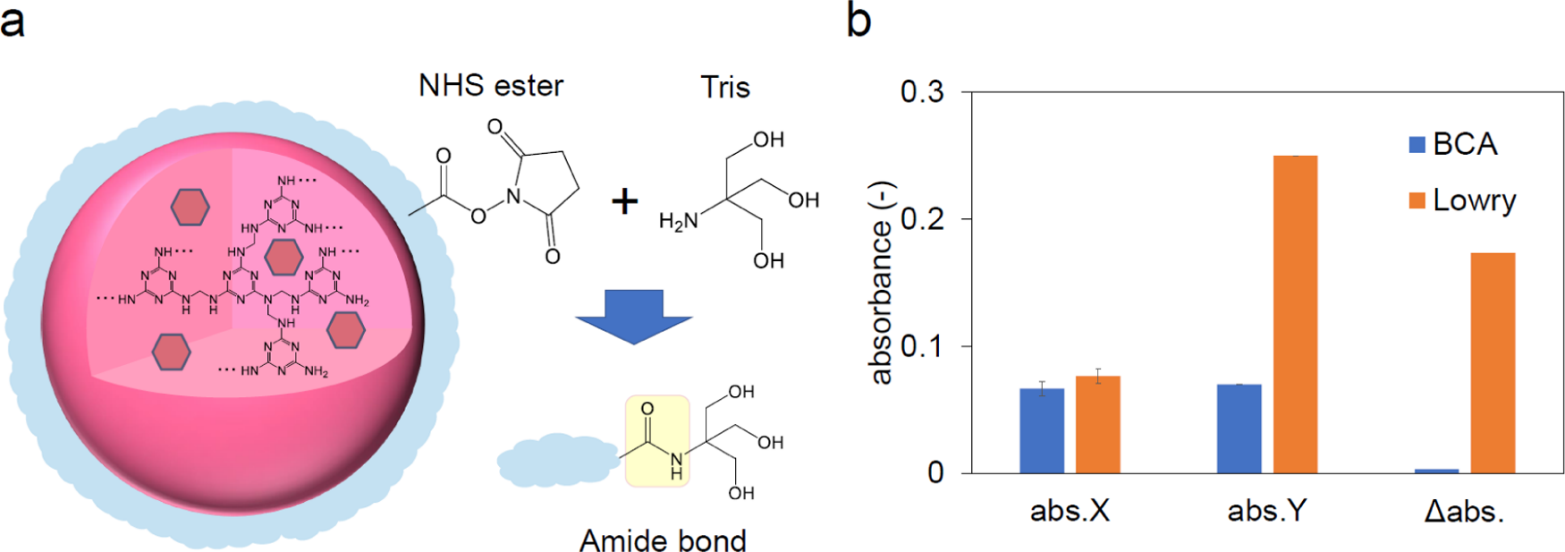
(a) Schematic of the formation of amide bonds by coupling between NHS ester and Tris. (b) Absorbance before and after Tris addition (abs.*X* and abs.*Y*, respectively) and their difference (Δabs).

Three methods (BCA, Bradford, and Lowry) widely used for quantifying protein were employed to quantify the SA content binding to SA-CMD-MNPs. All methods showed high linearity in the calibration curves of protein concentration and absorbance based on each principle (Figure S4a–c). In the interference experiment, when NMPs were added to the SA solution, neither BCA nor Lowry showed an effect of less than 10% on SA absorbance (Figure S4d,e), but when MNPs were added to Coomassie Brilliant G-250 solution in Bradford, a blue precipitate formed immediately (Figure S4f). Since Coomassie Brilliant G-250 mainly utilizes basic (Arg, Lys, His) and aromatic (Trp, Phe, Tyr) amino acid residues of protein to form complexes,^51^ we infer that the primary or secondary amino groups of melamine were involved in the complex formation. Finally, we confirmed the false positives for SA against the amide bond formed between CMD and Tris, and there was no difference in the absorbance of BCA when Tris was bound to the NHS ester residue of CMD by amide coupling, whereas Lowry showed an increase in absorbance (Figure 6).

Although not studied in detail herein, the Folin–Ciocalteu reagent, Lowry’s chromogenic source, reacts with various compounds other than proteins,^52,53^ and even compounds with only one amide bond, such as acetohydroxamic acid (H_3_CC(O)NHOH), are chromogenic.^52^ We infer that this compound was the source of coloration in this study, although its structural formula is different from the (−CH_2_C(O)NHC(CH_2_OH)_3_) obtained from the Tris quenching of the NHS ester of CMD.

**Figure 7:**
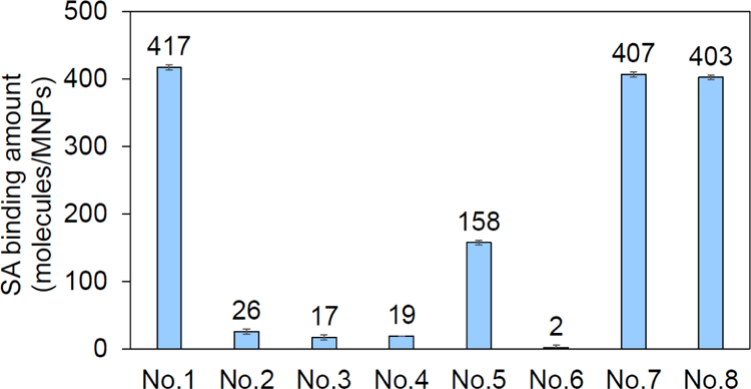
Amount of SA binding of SA-CMD-MNPs using the bicinchoninic (BCA) assay. Nos. 1–8 indicate SA-CMD-MNPs synthesized under the conditions shown in Figure 5A.

Figure 7 shows the amount of SA binding of the SA-CMD-MNPs characterized by the BCA assay.

When SA-CMD-MNPs were prepared by reacting MNPs with CMD, NHS, and EDC in an MES buffer, the SA binding amount per particle was 417 ± 4 (Figure 7). The same trend was obtained when the SA reaction buffer or the wash buffer after the SA reaction was changed to a phosphate buffer (Figure 7). When one of CMD, NHS, or EDC was absent, little SA binding was observed, indicating that the three compounds are essential for dispersion via CMD binding and functionalization via SA binding. As aforementioned, changing the buffer from MES to phosphate buffer during the CMD/NHS/EDC reaction decreased the reaction efficiency considering the ζ-potential (Table 2), and the decrease in the amount of SA binding can also be attributed to the decrease in the amount of CMD binding and the number of NHS esters present after the CMD reaction (Figure 7).

### IHC using SA-CMD-MNPs

**Figure 8:**
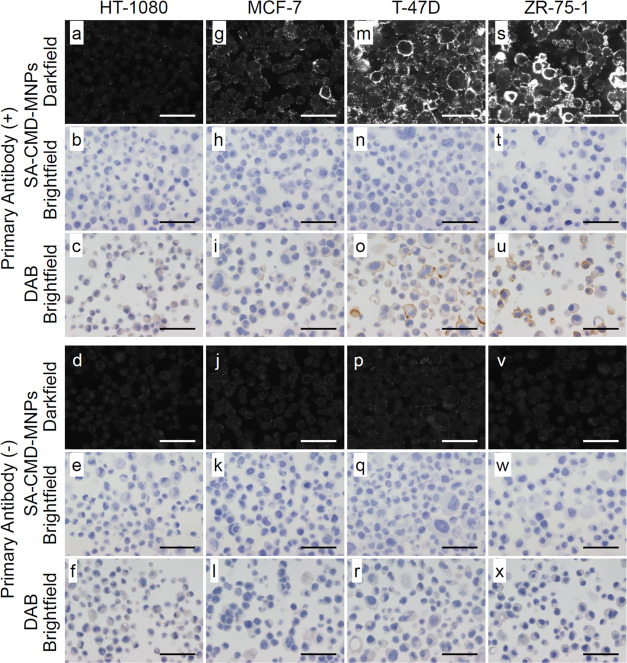
Immunohistochemistry (IHC) images of (a–f) HT-1080, (g–l) MCF-7, (m–r) T-47D, and (s–x) ZR-75-1. Four cultured cells were immunostained by SA-CMD-MNPs ((a, b, d, e) HT-1080, (g, h, j, k) MCF-7, (m, n, p, q) T-47D, (s, t, v, w) ZR-75-1) or DAB, ((c, f) HT-1080, (i, l) MCF-7, (o, r) T-47D, and (u, x) ZR-75-1) with (upper half) and without primary antibodies (lower half). Scale bar = 50 μm.

Finally, the prepared SA-CMD-MNPs were evaluated by IHC (Figure 8).

ZR-75-1, T-47D, MCF-7, and HT-1080 are human epithelial cells. ZR-75-1, T-47D, and MCF-7 are derived from breast cancer cells, and HT-1080 is derived from fibrosarcoma. HER2 expression levels of ZR-75-1, T-47D, MCF-7, and HT-1080 were 6.4 × 10^4^, 3.2 × 10^4^, 1.5 × 10^4^, and 2.3 × 10^3^ (HER2 molecules/cell), respectively.^54−56^ The expression levels of HER2 and the fluorescence intensity of the MNPs were also altered and localized to the plasma membrane, suggesting that the SA-CMD-MNPs were specifically bound to biotin-modified secondary antibodies on the cultured cells. In the absence of antibodies, the nonspecific adsorption of SA-CMD-MNPs was negligible, indicating that the nanoparticles have a high nonspecific adsorption capacity. Interestingly, nanoparticles prepared by washing the SA-CMD-MNPs with a phosphate buffer (pH 8.5) instead of Tris showed nonspecific adsorption on the nuclei (Figure S5), which may be attributed to the hydrolysis of NHS ester in the alkaline phosphate buffer instead of quenching, thereby exposing the carboxyl group. This shows the importance of masking the carboxyl group using a primary amine, such as Tris. Four different cell lines were immunostained by commonly used 3,3′-diaminobenzidine (DAB) to compare with SA-CMD-PIDs. The images immunostained by SA-CMD-PIDs resembled DAB images. Furthermore, the fluorescence derived from the SA-CMD-PIDs localized to the plasma membrane better than the chromogen derived from DAB in MCF-7, a cell line with low HER2 expression. This suggests that SA-CMD-PIDs are comparable to or better than existing methods.

## Conclusions

In this study, we found that CMD can be coupled to MNPs in a single step in the presence of NHS and EDC. CMD-MNPs conjugated with CMD via amide coupling showed excellent dispersibility, and SA could conjugate with MNPs without the addition of extra reagents by utilizing the excess NHS ester that was not used for the MNP-CMD coupling. SA-CMD-MNPs and CMD-MNPs showed excellent dispersibility and long-term stability, and the amount of protein bound by SA-CMD-MNPs could be quantified by the BCA assay, which showed that not only CMD but also NHS and EDC are essential for SA binding. This study shows that SA-CMD-MNPs can be used for biosensing and can specifically stain even HER2-low expressing cell lines, such as MCF-7. These results indicate that SA-CMD-MNPs are good biosensors. Finally, although MNPs were investigated in this study, the established method is applicable to other nanoparticles bearing amino groups, and this study would contribute to the development of MNPs.

## Supporting Information Available

SEM images of the CMD-MNPs and SA-CMD-MNPs; excitation and emission spectra of Lumogen F Red 305 and MNPs, CMD-MNPs, and SA-CMD-MNPs; FT-IR spectra and spectral band assignments of MNPs, CMD-MNPs, and SA-CMD-MNPs; calibration curves of protein concentration and absorbance; the interference of MNPs in BCA, Lowry, or Bradford; IHC images stained using SA-CMD-MNPs prepared by washing with phosphate buffer instead of Tris (PDF)
