# Supplementary material for: Water-Dispersible Carboxymethyl Dextran-Coated Melamine Nanoparticles for Biosensing Applications
Source: ACS Omega. 2022 Nov 3;7(45):41641–50. doi: 10.1021/acsomega.2c05653 (PMC9670359; doi:10.1021/acsomega.2c05653)
Supplement: Supplementary file 1 — ao2c05653_si_001.pdf [file ao2c05653_si_001.pdf]

Supporting Information

Water-Dispersible Carboxymethyl Dextran-Coated Melamine Nanoparticles for Biosensing Applications

Yoshikazu Kurihara,* Hiroyuki Yokota, and Masaru Takahashi

KONICA MINOLTA, INC. 1 Sakura-machi, Hino-shi, Tokyo 191-8511, Japan

Corresponding author Email: yoshikazu.kurihara@konicaminolta.com

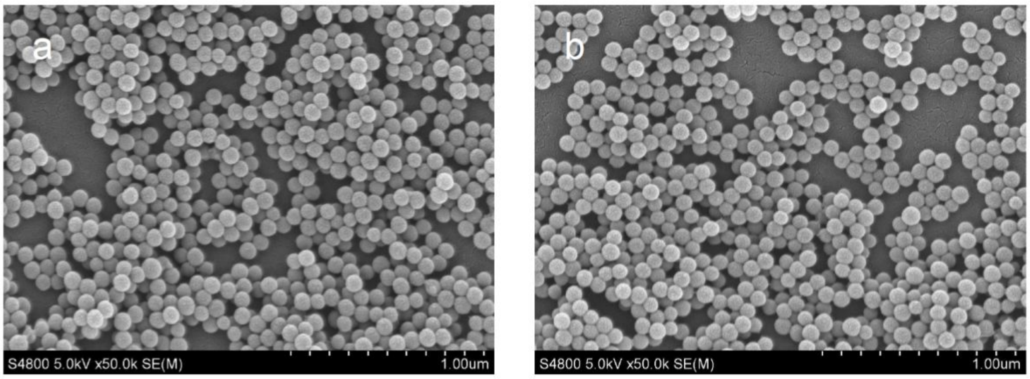

Figure S1. SEM images of (a) CMD–MNPs and (b) SA–CMD–MNPs.

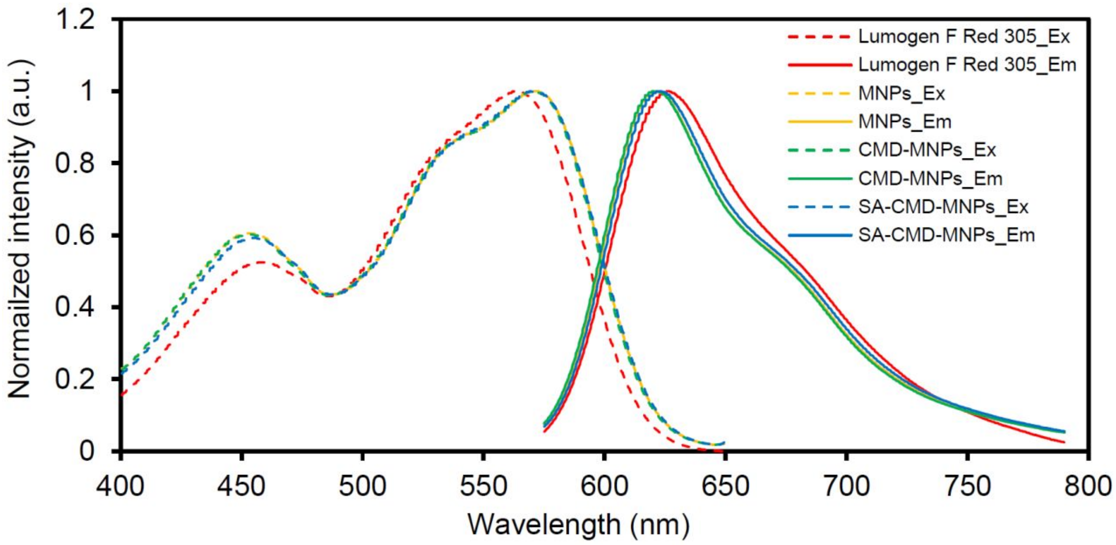

Figure S2. Excitation (Ex: dotted line) and emission (Em: solid line) spectra of Lumogen F Red 305, MNPs, CMD–MNPs, and SA–CMD–MNPs.

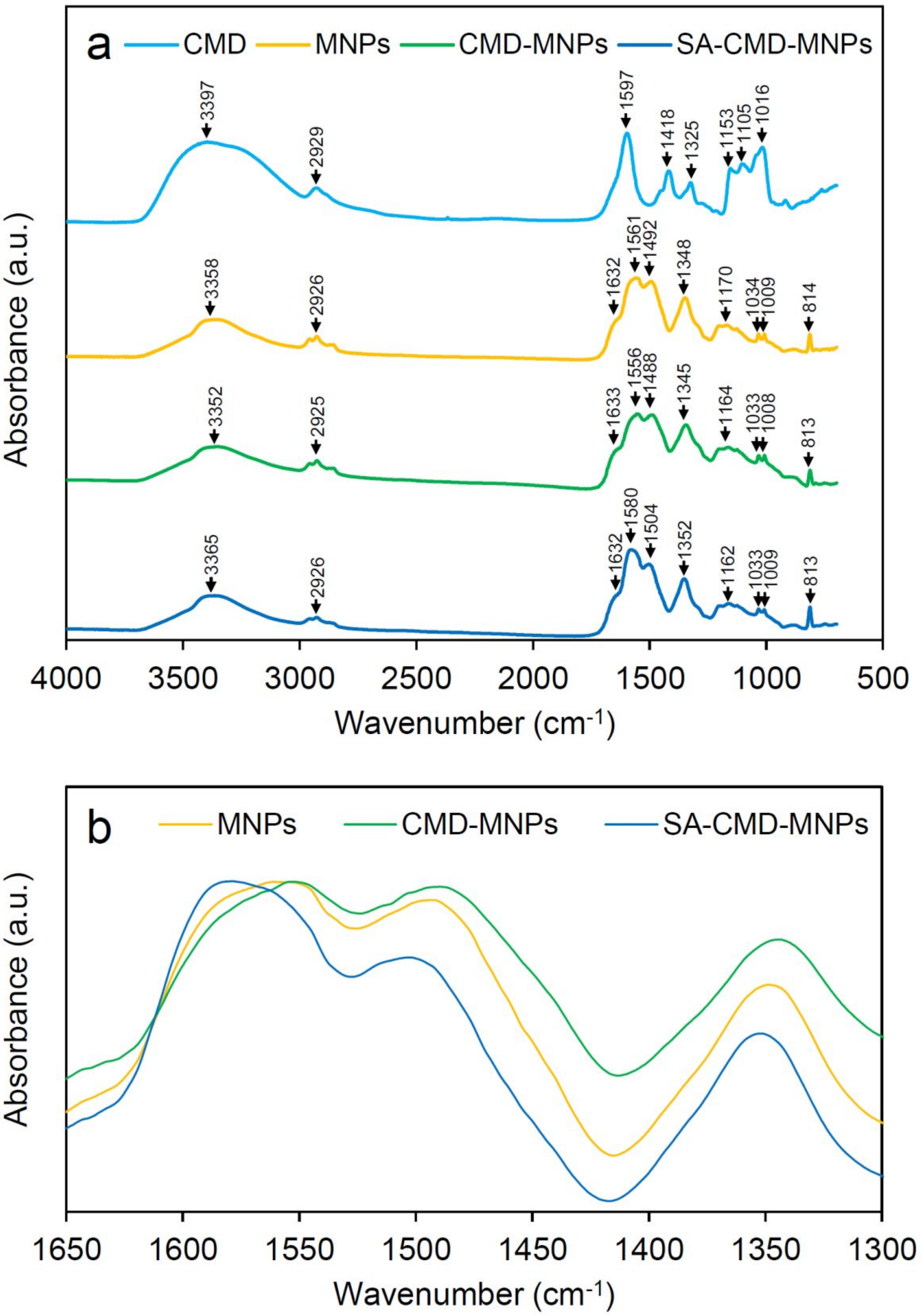

Figure S3. FT–IR spectra of (a) CMD, MNPs, CMD–MNPs, and SA–CMD–MNPs (700–4000 cm^{-1}), (b) MNPs, CMD–MNPs, and SA–CMD–MNPs (1300–1650 cm^{-1}).

Table S1. FT-IR spectral band assignments of MNPs, CMD–MNPs, and SA–CMD–MNPs.

Assignment		Vibration	MNPs (cm^{-1})	CMD-MNPs (cm^{-1})	SA-CMD-MNPs (cm^{-1})
C=N	Triazine	Bending	814	813	813
C-H	Methylol melamine	Bending	1009	1008	1009
C-O-C	Methylol melamine	Stretching	1034	1033	1033
C-N	Between methylol group and triazine ring	Stretching	1170	1164	1162
C-H	Methylene	Bending	1348	1345	1352
C-H	Methylene	Bending	1492	1488	1504
C=N	Triazine	Stretching	1561	1556	1580
N-H	Melamine	Bending	1632	1633	1632
C-H	Methylol melamine	Stretching	2926	2925	2926
N-H	Methylol melamine	Stretching	3358	3352	3365

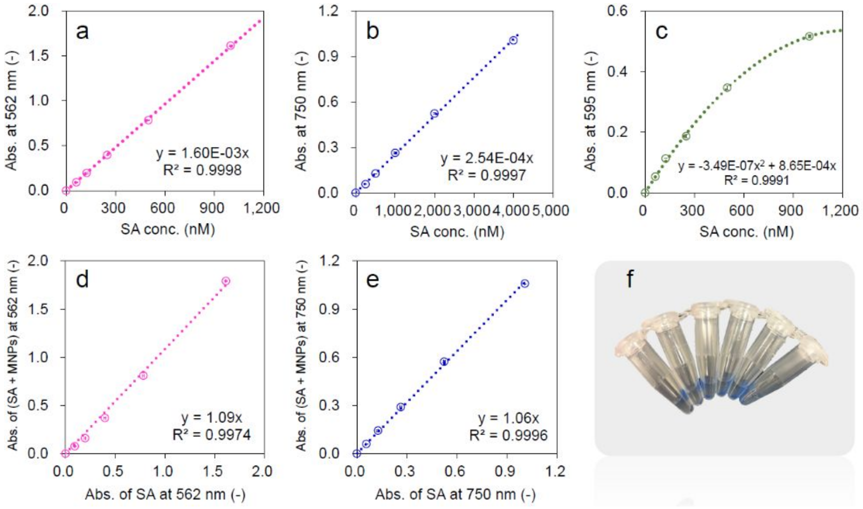

Figure S4. (a−c) The calibration curves of protein concentration and absorbance based on (a) BCA, (b) Bradford, or (c) Lowry. (d−f) Interference of MNPs in (d) BCA, (e) Bradford, or (f) Lowry. When MNPs were added to Coomassie Brilliant G-250 solution, a blue precipitate formed immediately (f).

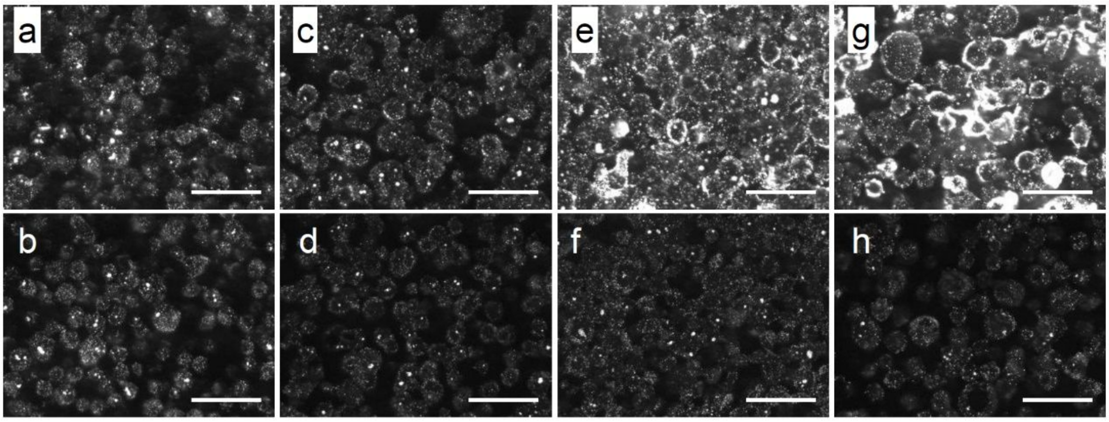

Figure S5. Immunohistochemistry (IHC) images of (a, b): HT1080, (c, d): MCF-7, (e, f):

T-47D, (g, h): ZR-75-1. Four cultured cells were immunostained with (upper row) and without primary antibodies (lower row) using MNPs prepared by washing SA–CMD–MNPs with a phosphate buffer instead of Tris. Scale bar = 50 μm.
